# *Origanum vulgare* L. extract-mediated synthesis of silver nanoparticles, their characterization and antibacterial activities

**DOI:** 10.1186/s13568-020-01100-9

**Published:** 2020-09-05

**Authors:** Syuzanna Hambardzumyan, Naira Sahakyan, Margarit Petrosyan, Muhammad Jawad Nasim, Claus Jacob, Armen Trchounian

**Affiliations:** 1 Department of Biochemistry, Microbiology and Biotechnology, Biology Faculty, Yerevan State University, 1 Alex Manoogian Str, 0025 Yerevan, Armenia; 2 Division of Bioorganic Chemistry, School of Pharmacy, Saarland University, Saarbrucken, 66123 Germany

**Keywords:** *Origanum vulgare* L., Leaf extract and phenol content, Antioxidant activity, Plant extract mediated synthesis of silver nanoparticles, Antibacterial activity

## Abstract

Plant extracts serve as reducing and coating agents and are, therefore, commonly employed for the generation of silver (Ag) nanoparticles (NPs). Plant extract mediated synthesis of Ag NPs is a green, environmentally friendly and cost-effective technique which offers a new and potential alternative to chemically synthesized NPs, decreasing the utilization of hazardous and toxic chemicals and protecting the environment. *Origanum vulgare* L. extracts were evaluated for total flavonoid and phenol content. The free radical scavenging activity was determined employing 2,2-diphenyl-1-picrylhydrazyl assay. Ag NPs were produced exploiting ethanolic extracts of *O. vulgare* L. leaves. The generation of Ag NPs was carried out both in light and dark conditions. The biosynthesized Ag NPs were characterized employing microscopic and spectroscopic techniques. Antibacterial activities of Ag NPs were determined following appropriate methods. The results revealed that energy of photons was required to reduce Ag^+^ to Ag^0^. According to scanning electron microscopy reports, biologically formed Ag NPs ranged in size from 1 to 50 nmand were presented instability causing aggregation. They indicated that *O. vulgare* L. extracts were rich in flavonoids and phenols and exhibited strong antioxidant activity. Ag NPs exhibited good antibacterial activity immediately after production. Gram-positive strains showed higher sensitivity to Ag NPs compared to Gram-negative stains. Ag NPs can serve as an effective antibacterial agent against antibiotic-resistant strains. The kanamycin-resistant strain was more sensitive to Ag NPs than the ampicillin-resistant strain. Thus, Origanum extract-mediated synthesized Ag NPs can be recommended as alternative effective antibacterial agents, but their activity depended on bacterial species and strains.

## Key points

1. Biosynthesis of quality Ag nanoparticles (NPs) using *O. vulgare* L. leaf extracts under light condition.
2. Significant inhibition of the growth of Gram-positive, Gram-negative bacteria and antibiotic-resistant strains by Ag NPs exploited.

## Introduction

The emergence of resistance to currently available antibiotics is a huge challenge faced by the humanity. According to estimates by the European Center for Disease Prevention and Control, 25,000 people die in Europe from drug-resistant bacterial infections every year. The most recent pandemic Covid-19 situation which has affected more than twenty million people across the globe until now is the latest example which highlights the need to develop cure against these species. The emergence of resistance has, therefore, provoked the need to develop alternative agents against the pathogenic bacterial strains (Trchounian et al. 2018; Kraemer et al. 2019).

Silver (Ag) is a unique multi-purpose element which was commonly exploited to prepare coins and jewelry. Evidence of the antibacterial properties of silver also dates back to ancient times, as testified by various historical stories well known to the public (Alexander, 2009). In recent years, the utilization of Ag nanoparticles (NPs) for medical purposes has attracted great attention of researchers because of their exceptional antimicrobial activities especially against a wide range of pathogenic microorganisms (Srirangam and Rao 2017; Trchounian et al. 2018).

Generally, Ag^+^ is a notorious toxic ion which causes oxidative stress and damages in various cellular components, including DNA, proteins, and the cell membrane. Elemental Ag NPs, in contrast, serve as an interesting dosage form characterized by a slower dissolution rate, which provides continuous intracellular supply of Ag^+^ leading to the death of target microorganisms (Mcshan et al. 2014) although some authors conclude that the mechanisms of action of Ag^+^ and Ag NPs may be not identical (Anna et al. 2018).

There are several methods for the generating Ag NPs, including physical grinding, wet-chemical reactions and biological methods. Physical methods usually generate very stable and small Ag NPs in high concentrations. The physical methods are, however, generally associated with numerous disadvantages. The most promising disadvantage includes the irreversible harm to the environment since such methods generally consume substantial amount of energy (Dhand et al. 2015). Similarly, the chemical methods also pose severe burden to the environment. Reagents frequently employed in chemical methods, such as reducing agents (sodium borohydride and hydrazine), stabilizers (for example, thiols, acids, alcohols) and solvents are inherently hazardous and harmful to the environment (Kondeti et al. 2017). The biological methods, in contrast, are rather simple, fast, inexpensive, energy efficient and do not threaten the environment (Tarannum and Gautam 2019). Ag NPs are biologically synthesized exploiting various microorganisms (yeast, fungi and bacteria) and extracts of plant tissues (leaves, roots, fruits, stems and flowers etc.). Among biological methods, plant extract-based production of Ag NPs seems more justified. In addition to other advantages of the synthesis of Ag NPs mediated by the plant extracts, secondary metabolites present in the plant extracts as coating agents with antioxidant activity may reduce the side effects of Ag NPs, making them more suitable for medical applications (Shaik et al., 2018; MoghrovyanA et al. 2019; Aghajanyan et al., 2020). Plant extracts contain various natural phytochemicals, such as water-soluble flavonoids and polyphenolic compounds, which serve as antioxidants. These phyto-molecules are strongly reducing in nature and easily adsorb at the surface of NPs, thereby enhancing their stability (Salari et al. 2019). In addition, plant-based antioxidants prevent the oxidation of Ag atoms (Ag^0^) at the surface of NPs by forming a protective coating around the particles and thereby avoiding direct interaction with molecular oxygen. If these coating components are not firmly adhered to the surface of the NPs and displaced by solvent molecules, the physical and chemical stability of NPs is adversely affected leading to aggregation or oxidation (Mcshan et al., 2014; Aghajanyan et al. 2020). *O. vulgare* L., belonging to the *Lamiaceae* family as a major source of natural secondary metabolites with redox properties, can be effectively used for the synthesis of Ag NPs (MoghrovyanA et al. 2019).

In the current study, a culinary herb *O. vulgare* L. was collected from four different provinces of Armenia *i.e.* Kotayk, Lori, Tavush, Gegharkunik, and its ethanolic extract was employed for green synthesis of Ag NPs. The ethanolic extract of *O. vulgare* L. is rich in polyphenols, which contribute to its reducing potential. The biosynthesized Ag NPs were characterized and quantified by several techniques, such as UV–Vis spectrophotometry, Dynamic Light Scattering (DLS) and Laser Diffraction (LD), Scanning Electron Microscopy (SEM) couple with Energy Dispersive X-ray (EDX) analysis, Inductively Coupled Plasma-optical emission spectrometry (ICP-OES). Moreover, the potential antibacterial properties of Ag NPs were evaluated against various Gram-positive (*Staphylococcus aureus, Bacillus subtilis*), Gram-negative bacterial strains (*Escherichia coli*, *Salmonella typhimurium*), ampicillin-resistant *E. coli*, and kanamycin-resistant *E. coli* stains. The growth kinetics of *E. coli* when exposed to plant extract and Ag NP were also studied.

## Materials and methods

### Plant materials and preparation of *O. vulgare* extracts

The plant material (*O. vulgare* L.) was collected from four provinces of Armenia; Kotayk (OV 1), Lori (OV 2), Tavush (OV 3), Gegharkunik (OV 4) during bloom in July 2016. The identification of plant samples was carried out at the Department of Pharmacognosy, Yerevan State Medical University, Yerevan (Armenia). Plant samples were deposited and are available at the Herbarium of the Institute of Botany, National Academy of Sciences of Armenia, Yerevan, Armenia (Voucher specimen number: ERE191395). The collected leaves were washed, dried in the shadow at room temperature and subsequently crushed to obtain powder which was stored in a dry and dark place at room temperature until use.

1 g of *O. vulgare* leaves powder was weighed and suspended into 10–15 mL of 40% ethanol and stirred overnight at ~ 10 °C. The extract was subsequently centrifuged for 5 min at 5000 rpm, and the supernatant was isolated. The precipitate was extracted four times with the same procedure, and the combined supernatant fractions were evaporated at room temperature to obtain dried extracts which were then stored at 4 °C until further use.

### Determination of total flavonoid content

The total flavonoid content of *O. vulgare* plant extracts was determined employing AlCl_3_ colorimetric assay (Ghasemi et al. 2009). The extract was dissolved in 80% ethanol to obtain a final concentration of 1 mg mL^−1^. 0.5 mL of this extract solution was mixed with 0.1 mL of AlCl_3_ (10%), 0.1 mL of sodium acetate (1 M) and 2.8 mL of distilled water. The sample was incubated for 15 min and the absorbance of the samples was measured at 415 nm against a blank consisting of distilled water utilizing a UV–Vis spectrophotometer (Genesys 10S, Thermo Scientific, USA). Total flavonoid content was determined employing a calibration curve of quercetin (Q), as a reference flavonoid (0–1000 µg mL^−1^) and results were expressed in terms of Q equivalents (QE) per g extract dry weight.

### Determination of total phenolic content

The total phenolic content of plant extracts was measured exploiting the Folin–Ciocalteu (FC) reagent according to the method described by Genwali et al. (2013). The plant extract was dissolved in distilled water to obtain a final concentration of 1 mg mL^−1^. 0.5 mL of plant extracts solution was mixed with 0.1 mL of FC reagent and incubated for 7 min at room temperature followed by the addition of 1 mL of Na_2_CO_3_ solution (7%) and 0.9 mL of distilled water. The mixture was shaken thoroughly and kept in the dark at room temperature for 1 h. The absorbance of samples was measured at 765 nm using a UV–Vis spectrophotometer (Genesys 10S, Thermo Scientific, USA). Distilled water was exploited as a blank. Total phenolic content was determined employing a calibration curve of gallic acid (GA) (0–250 µg mL^−1^). The total phenolic content was expressed in terms of GA equivalents (GAE) per g extract dry weight.

### 2,2-Diphenyl-1-picrylhydrazyl free radical scavenging assay

Free radical scavenging activity of different *O. vulgare* leave extracts was measured by 2,2-Diphenyl-1-picrylhydrazyl (DPPH)—free radical scavenging assay (Xu et al. 2011). Various concentrations of plant extracts (ranging from 31.25 to 500 µg mL^−1^) were prepared by dilution method 0.0.1 mM DPPH was prepared in 96% ethanol. 200 µL of aqueous plant extracts of different concentrations were added to 1 mL of DPPH solution. The mixture was shaken vigorously and allowed to stand in dark condition at room temperature for 30 min. The absorbance was measured at a wavelength of 517 nm against a blank (96% ethanol) using a UV–Vis spectrophotometer (Genesys 10S, Thermo Scientific, USA). Catechin was used as a positive control under the same assay condition. The radical scavenging activity was calculated employing the following equation:

$$ {\text{Radical scavenging activity}}\left( {\text{\% }} \right) = \left[ {\frac{{\left( {Abc - Abs} \right)}}{Abc}} \right] \times 100 $$

where Abc and Abs represented the absorbance of the control (DPPH solution alone) and the absorbance of the sample in the presence of an extract or standard, respectively. The results were expressed as IC_50_ values (µgmL^−1^), which represented the concentration of sample required to inhibit 50% of the DPPH free radicals.

### Synthesis of Ag NPs using *O. vulgare* extracts

Stock solution of *O. vulgare* extract was prepared by dissolving 5 mg of plant extract in 10 mL of Milli-Q water (18.2 MΩ·cm at 25 °C). Ag NPs were synthesized by mixing the solutions of AgNO_3_ (10 mM) and plant extract (0.5 mg mL^−1^) in 1:9 ratio to achieve a final concentration of 1 mM for AgNO_3_ (Rautela et al. 2019). A control sample excluding plant extract was also prepared similarly. The samples were agitated on a shaker with constant rotation (150 rpm) under dark (no light) and light (normal room light) conditions at a temperature of 23 ± 2 °C for 18 h. For dark conditions, the tubes were wrapped with aluminum foil to protect from light (Srikar et al. 2016).

### Characterization of biosynthesized Ag NPs

The optical properties of Ag NPs were characterized exploiting a UV–Vis spectrophotometer (Lambda 35, Perkin Elmer) with a slit width of 1 nm of slit width and at a scan speed of 480 nm min^−1^. The analysis was performed employing quartz cuvettes over a path-length of 10 mm. The absorption of samples was measured in the wavelength range (λ) of 350–700 nm. The device was equipped with the UV WinLab software package.

The physical stability of the samples was evaluated by measuring the zeta potential (ξ-potential) and dynamic light scattering (DLS). The ξ-potential and DLS measurements of the samples were performed employing a Zeta-sizer Nano ZS (Malvern Instruments, UK) which analyzed the electrophoretic mobility of the samples in an electric field. The size of biosynthesized Ag NPs was imaged by SEM (ZEISS-SUPRA 40/gemini column) equipped with Electron Backscatter Diffraction (EBSD) detector and Energy Dispersive X-ray Spectroscopy (EDS) detector -SEM–EDX analysis. The total Ag content was determined by inductively coupled plasma-optical emission spectroscopy (ICP-OES) (Horiba Jobin–Yvon Ultima 2)).

### Antibacterial activity of biosynthesized Ag NPs

The antibacterial activity of biosynthesized Ag NPs was evaluated against a plethora of microorganisms including, Gram-positive bacterial strains (*S. aureus* MDC 5233 (Microbial Depository Center, MDC (National Microbial Culture Collection, WDCM 803, National Academy of Sciences of Armenia, Yerevan, Armenia; laboratory control strain), *B. subtilis* WT-A17 (isolated from metal polluted soils of Kajaran, Armenia), Gram-negative bacterial strains *(E. coli* VKPM-M17 (Russian National Collection of Industrial Microorganisms at the Institute of Genetics and Selection of Industrial Microorganisms, Moscow, Russia; laboratory control strain)), *S. typhimurium* MDC 1754 (laboratory control strain)), ampicillin-resistant *E. coli* DH5α-pUC18 and kanamycin-resistant *E. coli* pARG-25 (supplied by Scientific-Production Center “ArmBiotechnology”, National Academy of Sciences of Armenia, Yerevan, Armenia) strains by disk diffusion method employing disks with 6 mm in diameter (Lawhavinit et al., 2010). The Mueller–Hinton agar was exploited for growth of bacteria. Disks immersed in plant extracts (0.5 mg mL^−1^), and Ag NPs solutions were placed separately on Petri dishes containing meat-peptone agar contaminated with bacteria. Distilled water was employed as a negative control whilst ampicillin (20 µg mL^−1^) was exploited as standard positive control for all cases excepted ampicillin-resistant *E. coli* DH5α-pUC18, for which kanamycin (20 µg mL^−1^) was utilized. The plates were then incubated for 18 h at 37 °C, and the diameters of bacterial growth inhibition zones were recorded in millimeter (mm). The antibacterial activity of biosynthesized Ag NPs was compared with colloidal Ag, commercially available under “Silverton” trademark (“Tonus-Les” Lab, Armenia), and produced by electrochemical process (Soghomonyan et al., 2019). The antibacterial activity of Ag NPs was also recorded in terms of Minimum Inhibitory Concentration (MIC) two-fold serial dilution method (110–6.875 µg mL^−1^). MIC values were taken after 18 h of incubation at 37 °C. MIC value was considered as the lowest concentration of NPs, which suppressed the growth of the test-bacterium (the concentration which form the smallest inhibition zone around the disk).

### Growth kinetics of *E. coli* VKPM-M17 under the influence of biosynthesized Ag NPs

The growth kinetics assay was employed to understand the pattern of bacterial growth under the influence of biosynthesized Ag NPs. The growth kinetics of *E. coli* VKPM-M17 was also monitored in the presence of OV 3 plant extract. Fresh *E. coli* colonies were isolated from meat-peptone agar plates and transferred to LB broth (pH 7.5) followed by incubation for 18 h at 37 °C. The antibacterial activity of Ag NPs was estimated in terms of MIC. The antibacterial effect of extract (OV3) was monitored at the concentration which was employed to produce Ag NPs *i.e.*0.5 mg mL^−1^. Bacterial growth curves were determined by measuring the turbidity of samples containing bacteria at 565 ± 15 nm every 30 min exploiting a densitometer (DEN-1B, BIOSAN, Latvia) (Szermer-Olearnik and Zwolińska 2014).

The growth rate constant (μ_1_) and mean generation time (g_1_) for *E. coli* VKPM-M17 were calculated during log growth phase (t_0_ = 0, t = 1.5 h) employing the following formulas:

$$ \mu \, = \,[\left( {{ \log }_{ 10} {\text{N }}{-}{ \log }_{ 10} {\text{N}}_{0} } \right)\, \times \, 2. 30 3]/\left( {{\text{t}}{-}{\text{t}}_{0} } \right) $$

$$ {\text{g}}\, = \,\left( {{ \log }_{ 10} {\text{N}}_{\text{t}} {-}{ \log }_{ 10} {\text{N}}_{0} } \right)/{ \log }_{ 10} 2 {\text{ or g}}\, = \,0. 6 9 3/\mu $$

where N is the number of cells (Maclean et al. 2009).

### Data processing

The experimental measurements were expressed as average of three analyses calculated with ± SD employing Microsoft Office 365. The diameters of NPs were measured exploiting ImageJ software. Analysis of the data distribution was performed with the Student’s *t* test. P values of less than 0.05 were considered as statistically significant.

## Results

### The total flavonoid-phenolic composition of *O. vulgare* extracts

Flavonoids and other phenolic compounds are generally associated with redox and antioxidant properties.The total content of flavonoids in different *O. vulgare* extracts was determined from the calibration curve of Q (y = 2.3083x + 0.0369, R^2^ = 0.9947). A reasonable amount of flavonoid was found in all samples. The highest amount of flavonoid was observed in the sample collected from Tavush region (69.65 ± 1.09 mg (QE) g^−1^) followed by the sample collected from Gegharkunik region (53.54 ± 0.75 mg (QE) g^−1^). A rather decreased amount of flavonoid was observed in the sample collected from Lori region (41.63 ± 1.14 mg (QE) g^−1^) and the least quantity was found in the sample collected from Kotayk region (30.22 ± 0.25 mg (QE) g^−1^). Similarly, the total phenolic content of different *O. vulgare* extracts was calculated from a calibration curve of GA (y = 0.0063x + 0.0718, R^2^ = 0.9789). A reasonable number of polyphenols was observed in all samples. The highest content of polyphenols was observed in the sample collected from Tavush region (202.68 ± 1.27 mg (GAE) g^−1^) followed by the sample collected from Lori region (185.27 ± 2.05 mg (GAE) g^−1^). A rather lower content of polyphenols was observed in the sample collected from Kotayk region (165.01 ± 1.07 mg (GAE) g^−1^) and the least quantity was found in the sample collected from Gegharkunik region (150.19 ± 0.69 mg (GAE) g^−1^). The overall results indicate that the sample collected from Tavush region contains the highest content of polyphenols and flavonoid (Table 1).

**Table 1:** Total flavonoid and phenolic contents of *O. vulgare* extracts

Collection area	Extract name *O. vulgare*	Total flavonoid content, mg (QE) (g dry wt)^−1^	Total phenolic content, mg (GAE) (g dry wt)^−1^
Kotayk	OV 1	30.22 ± 0.25	165.01 ± 1.07
Lori	OV 2	41.63 ± 1.14	185.26 ± 2.05
Tavush	OV 3	69.64 ± 1.09	202.67 ± 1.27
Gegharkunik	OV 4	53.53 ± 0.75	150.19 ± 0.69

### Antioxidant activity of *O. vulgare* extracts

**Fig. 1:**
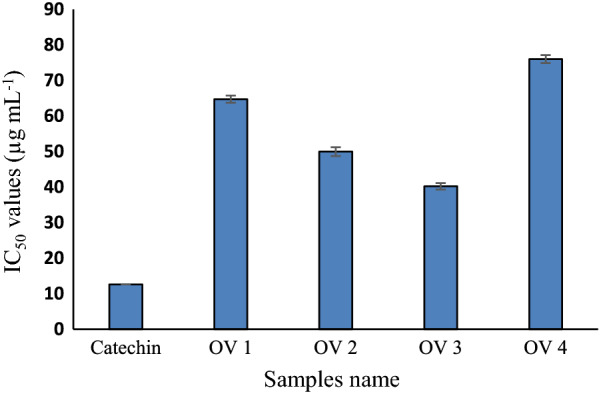
DPPH-free radical scavenging activity of *O. vulgare* extracts (OV 1, OV 2, OV 3 and OV 4) and catechin. The results are expressed in terms of IC_50_ values indicating the concentrations required to scavenge 50% of the free radicals

Since polyphenols and flavonoids are generally associated with radical scavenging activities, the four different *O. vulgare* extracts were, therefore, evaluated for DPPH-free radical scavenging activity and the results were expressed in terms of IC_50_ values (µg mL^−1^). All of the samples presented reasonable radical scavenging activities. The highest activity was observed for OV3 (40.22 ± 0.91 µg mL^−1^) followed by OV 2 (49.99 ± 1.24 µg mL^−1^). A relatively lower radical scavenging activity was observed for OV 1 (64.73 ± 1.01 µg mL^−1^) and the least activity was observed for OV 4 (75.99 ± 13 µg mL^−1^) (Fig. 1). Lower absorbance values of the reaction mixture indicated higher free radical activity. The antioxidant activity of plant extracts was compared with the antioxidant activity of catechin (12.62 µg mL^−1^), as positive control. The results are supported by several other numerous studies which confirm the notion that extracts with a high phenolic content exhibit strong antioxidant activity (Tungmunnithum et al. 2018).The highest activity was observed for OV3 (40.22 ± 0.91 µg mL^−1^),which could be associated with the highest amount of flavonoid and polyphenols.

### Generation and Characterization of Ag NPs

**Fig. 2:**
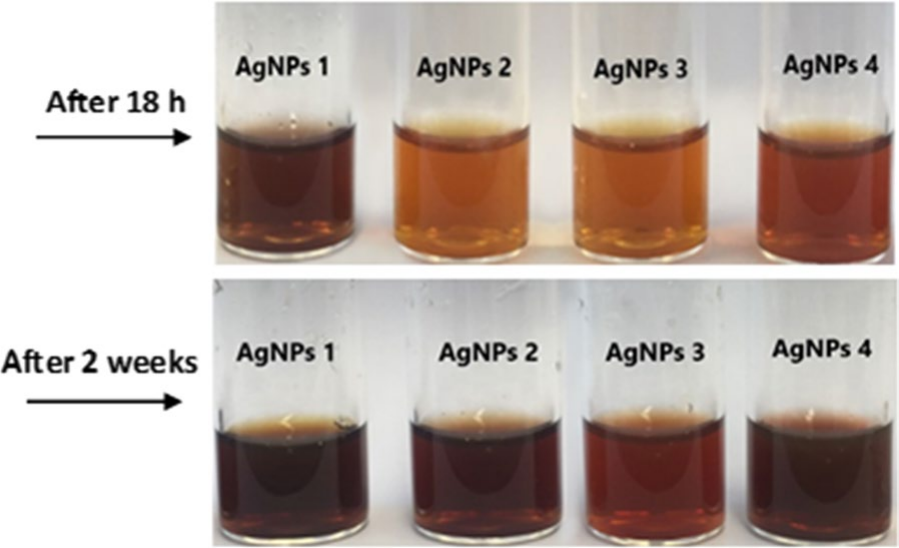
The nano-suspensions were analyzed for stability and change in color with the passage of time and the results confirm the unstable nature of the samples

Four groups of Ag NPs (*i.e.* Ag NPs 1, Ag NPs 2, Ag NPs 3 and Ag NPs 4) were produced exploiting OV 1, OV 2, OV 3 and OV 4 extracts, respectively. The formation of Ag NPs was confirmed by exploiting UV–Vis spectroscopy based analysis. In the presence of plant extracts, the color of the reaction mixture changed from light yellow to brown after 18 h of incubation. The previous literature about Ag NPs describes that the NPs are formed when color of the solution turns from light yellow to brown whilst a dark brown or black color indicates the possible oxidation of Ag NPs (Yong et al. 2013). The stability and shelf life of all nano-suspensions were investigated after keeping the samples in storage for more than two weeks after production. The results, however, pointed out that the samples were not very stable as indicated by the change in color of the samples (Fig. 2). No change in color was observed in the absence of plant extract, under similar conditions which confirm the notion that plant extracts are the key players for the generation of NPs.

**Fig. 3:**
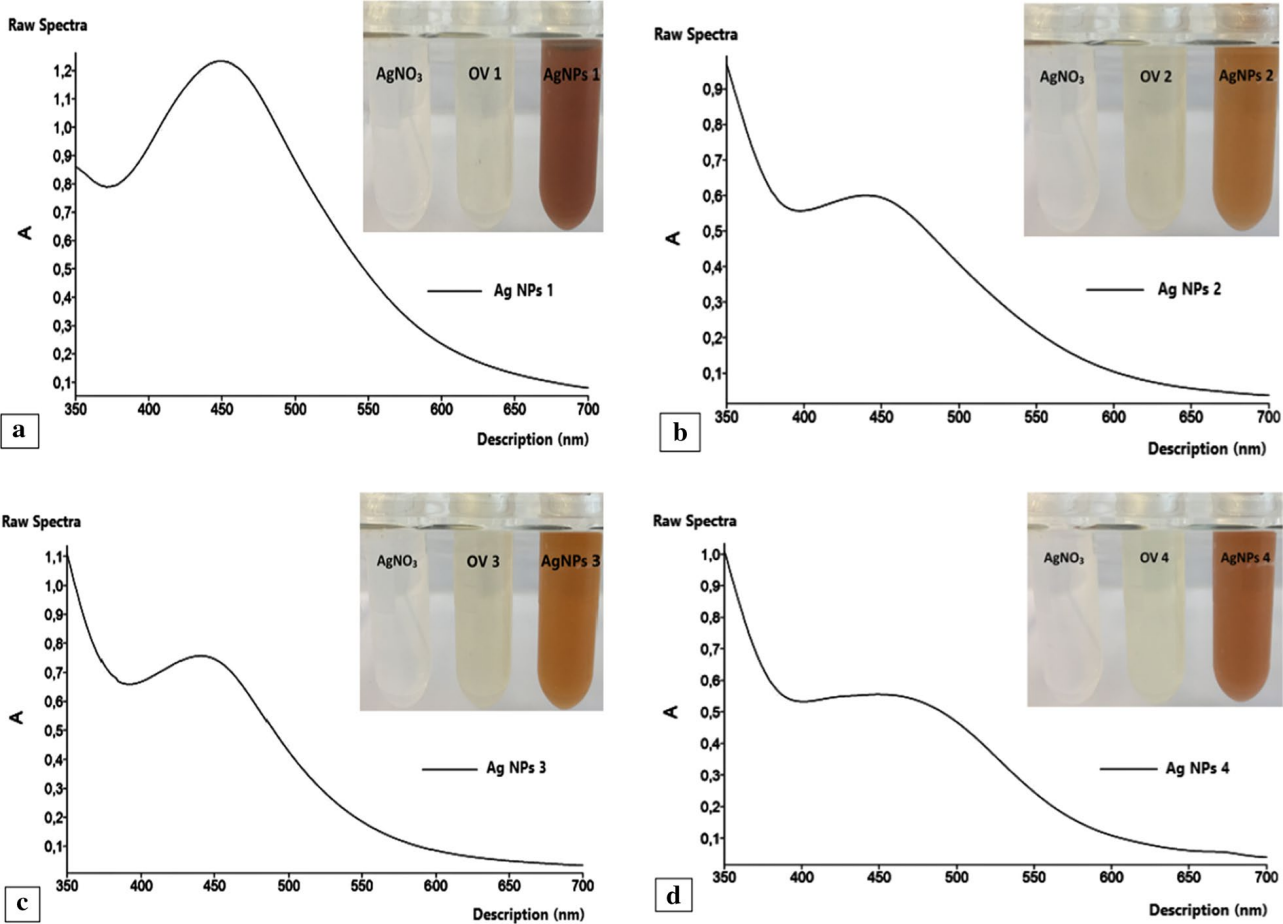
UV-Vis spectrophotometric analysis of Ag NPs 1 (**a**), Ag NPs 2 (**b**), Ag NPs 3 (**c**) and Ag NPs 4 (**d**). See the text

The samples were diluted 2.5 times for UV–Vis spectrophotometric analysis and the results indicated absorbance peaks around 440–460 nm which are specific for Ag NPs in the samples (Fig. 3). The results are in agreement with the literature data (Sankar et al. 2010; Sankar et al. 2014; Moodley et al. 2018; Behravan et al. 2019; Pirtarighat et al. 2019).

In the dark condition, the color of the Ag NPs reaction mixture did not change confirming the significance of light energy for the generation of Ag NPs (images not shown). The significance of light energy for the generation of NPs is also described by Rahman et al. (2019). Since the Ag NPs were not obtained in dark condition, the data described above only belongs to the samples exposed to light. Since Ag NPs belong to the category of plasmonic particles, they exhibit unusual optical properties. The electrons present in the conduction band at the surface of Ag NPs undergo collective oscillation when illuminated at specific wavelengths, a phenomenon known as surface plasmon resonance (SPR). SPR imparts high absorbing and scattering properties to the Ag NPs (Al-sharqi et al. 2019). The absorption peak of such particles may, therefore, shift depending on the size, shape and the environment surrounding the particles (Lee and Jun 2019). Previous studies have suggested that the SPR band shifts to a longer wavelength with increasing nanoparticle size known as redshift (Loiseau et al. 2019).

### Dynamic Light Scattering and Zeta Potential Studies

**Fig. 4:**
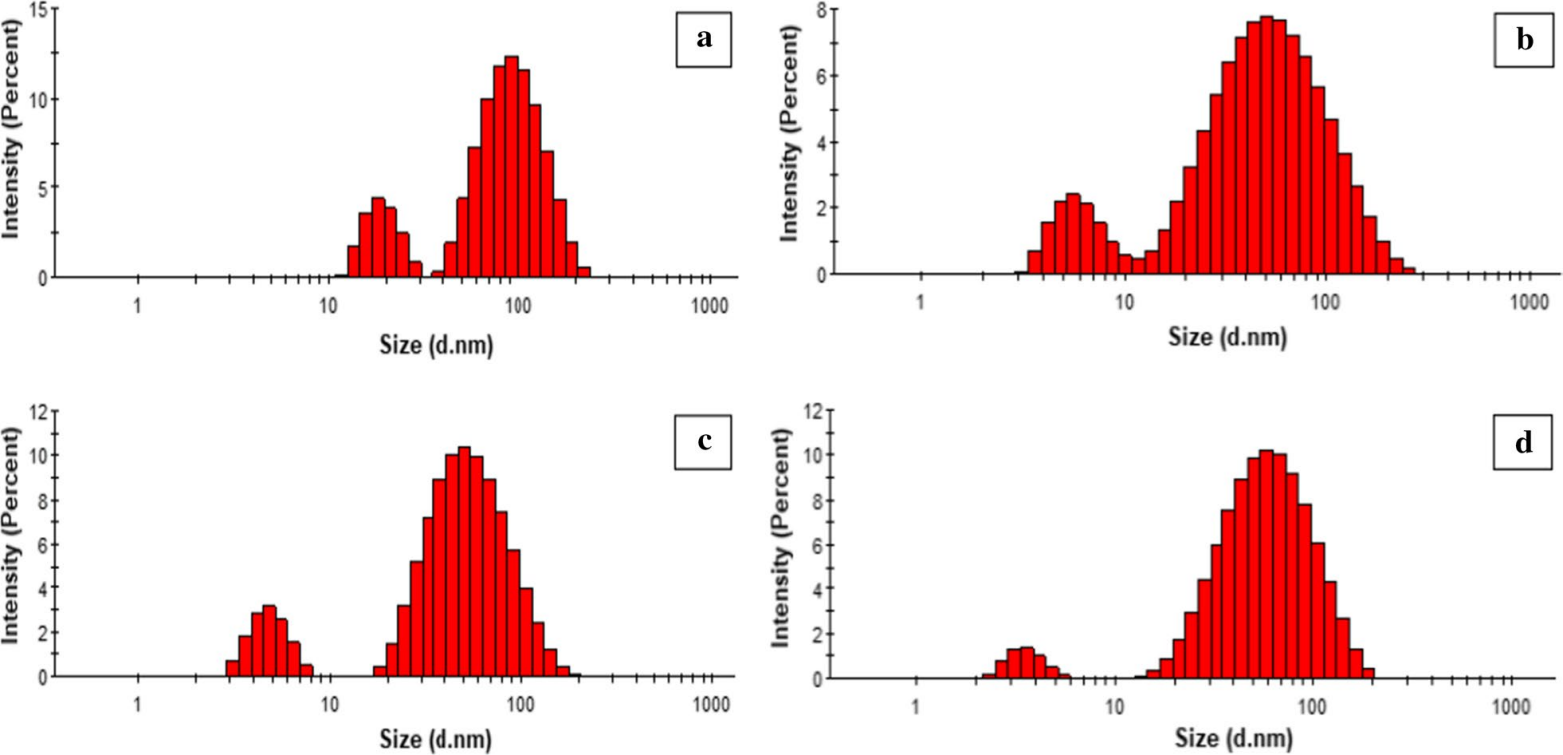
DLS analysis of Ag NPs: Ag NPs 1 (**a**), Ag NPs 2 (**b**), Ag NPs 3 (**c**) and Ag NPs 4 (**d**). See the text

DLS is a technique which measures the average particle size of nanoparticles in a sample. The principle of DLS operation is based on the method of laser beam diffraction. The incident light at the sample is mainly scattered by particles whose refractive index differs significantly from the solvent. The intensity of scattered light is detected by the DLS detector (Karmakar 2019). The results of DLS analysis revealed Z-average particle size of 58.81 ± 0.65 d.nm, 30.27 ± 0.17 d.nm, 30.20 ± 045 d.nm and 44.11 ± 0.56 d.nm for Ag NPs 1, Ag NPs 2, Ag NPs 3 and Ag NPs 4, respectively (Fig. 4). Polydispersity Index (PDI) is also an important indicator of quality in relation to size distribution. PDI values for the samples ranged from 0.291 to 0.536, which indicated that the samples are somewhat polydisperse in nature and the diameters of particle vary significantly (Danaei et al., 2018).

The ξ-potential is another significant parameter for understanding the surface charge and the tendency of aggregation of NPs. It is generally accepted that NPs with ξ-potential values of ± 0–10 mV, ± 10–20 mV, ± 20–30 mV, and ± 30 mV are unstable, relatively stable, moderately stable, and highly stable, respectively (Bhattacharjee, 2016). The arguments regarding the dependence of the stability of NPs on the ξ-potential differ slightly in the literature (Kumar and Dixit 2019). The ξ-potential values of -16.90 ± 1.13 mV, -21.30 ± 0.40 mV, -22.50 ± 0.55 mV and -26.70 ± 0.23 mV were recorded for the Ag NPs 1, Ag NPs 2, Ag NPs 3 and Ag NPs 4, respectively. NPs of these ξ-potential values are still prone to agglomeration, which may lead to further aggregation.

### SEM–EDX analysis

**Fig. 5:**
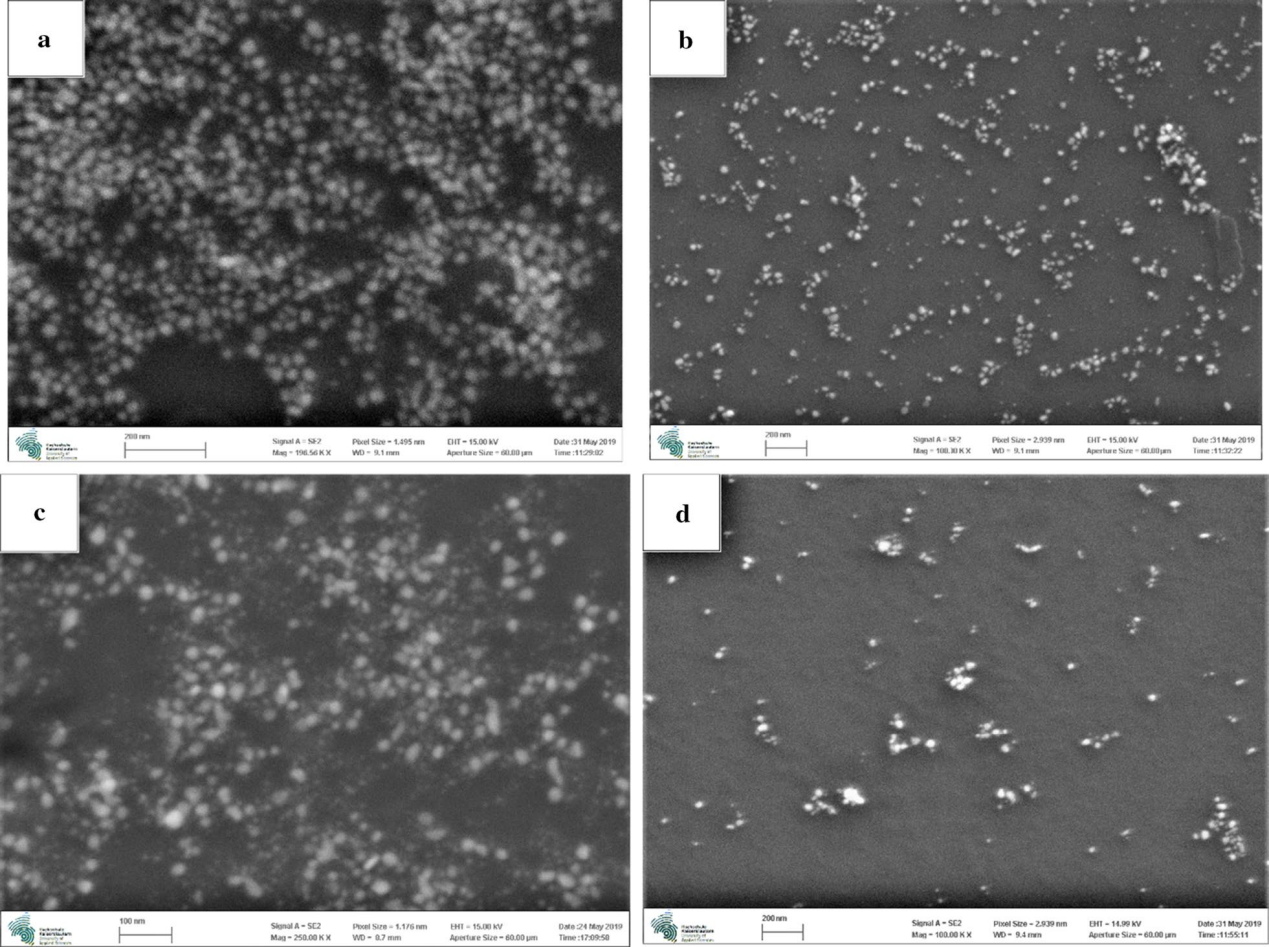
SEM analysis of Ag NPs: Ag NPs 1 (**a**), Ag NPs 2 (**b**), Ag NPs 3 (**c**), Ag NPs 4 (**d**). See the text

SEM was performed to determine the particle size distribution of Ag NPs. SEM images confirmed that that Ag NPs were obtained in small sizes ranging from 1 to 50 nm, as shown in Fig. 5. In the case of Ag NPs 2, Ag NPs 3 and Ag NPs 4 sizes of 1–25 nm prevailed, whilst in the case of Ag NPs 1, the predominant size was 30–50 nm.

EDX was employed to confirm the elemental composition of the reaction mixture (Menon et al. 2017). The EDX analysis presented the spectral signal in silver region confirming the presence of Ag NPs. The spectral signals of carbon, oxygen, chlorine, and sulfur were also observed, which could be associated to the presence of phytochemical constituents of plant extracts adsorbed at or near the surface of metal NPs.

### Inductively coupled plasma-optical emission spectroscopy analysis

The total Ag content was determined exploiting inductively coupled plasma-optical emission spectrometry (ICP OES). It is important to note that this method is not able to differentiate between Ag ions and Ag NPs and provides the cumulative content of both forms (Campos et al. 2017). Nevertheless, the analysis provides details about the quality of the samples. According to the results of ICP-OES Ag NPs were obtained in good amount. The highest quantity was achieved for Ag NPs 3 (110.1 ± 0.2 mg L^−1^) followed by Ag NPs 2 (107.3 ± 1.5 mg L^−1^). A slightly lower quantity was achieved for Ag NPs 4 (106.7 ± 1.1 mg L^−1^) and the least amount was observed for Ag NPs 1 (103.9 ± 0.8 mg L^−1^). The slight differences in the achieved quantities are of particular interest since the amount of AgNO_3_ initially employed for all the samples was same. These differences in quantities can be explained considering the operating principle of ICP-OES, where only small particles may be detected and large particles may go to waste. According to the ICP-OES, the lowest Ag concentration was achieved in case of Ag NPs 1, which indicates that this sample contained aggregated particles or Ag oxides, while the highest Ag content among all samples was achieved for Ag NPs 3, confirming the good quality of this sample.

### Antibacterial activity of Ag NPs

**Fig. 6:**
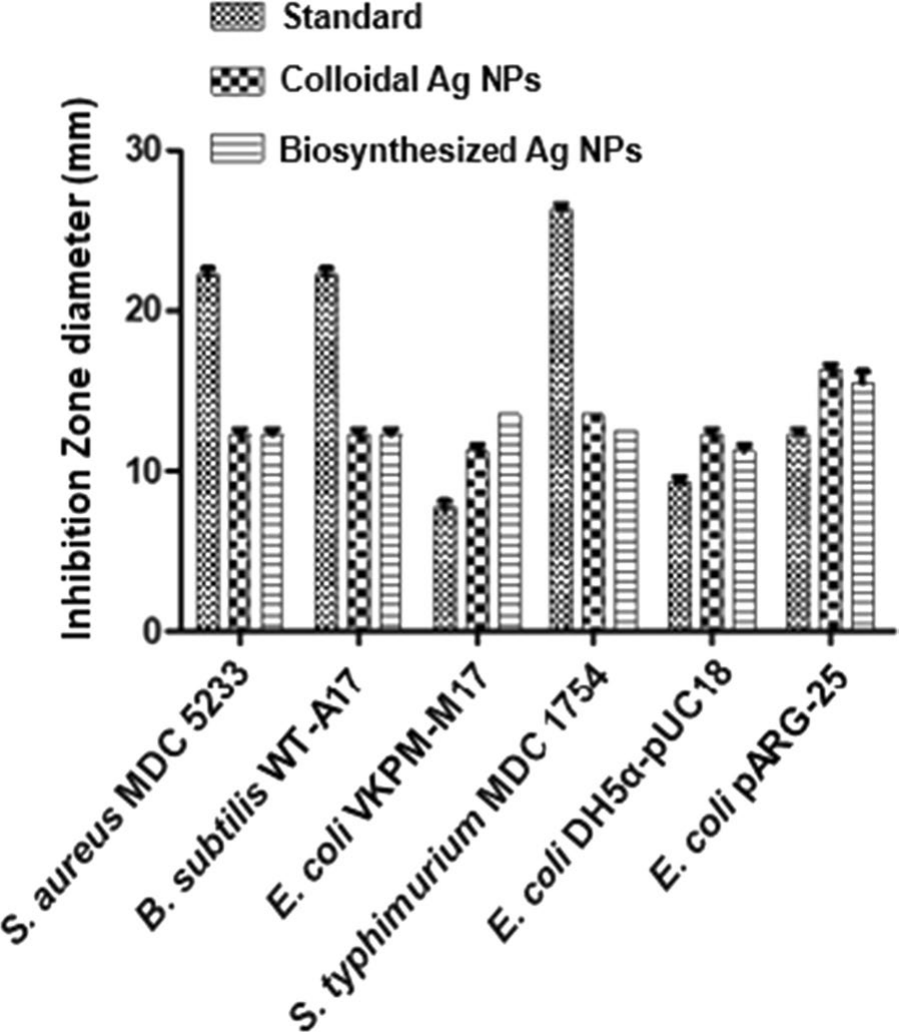
Antibacterial activity of the Ag NPs 3, colloidal Ag NPs and standards against the test bacteria. See the text

Based on the excellent results of characterization, Ag NPs 3 was selected for the antibacterial activity along with the relevant extract (OV3). The antibacterial activity was evaluated against several bacterial strains in terms of zone of inhibition and the antibacterial activity of Ag NPs 3 was compared with the activity of chemically synthesized colloidal Ag NPs (P > 0.05) (as reference sample) and standards (p < 0.05), as presented in Fig. 6. The plant extract alone did not exhibit marked antibacterial activity (data not shown). The antibacterial activity of biologically synthesized Ag NPs 3 in some cases was comparable to chemically produced colloidal Ag NPs at the same concentration.

To investigate the stability in the activity of Ag NPs the MIC values were presented, which were 13.75, 9.16, 18.35, 18.35, 18.35 and 11 µgmL^−1^ observed for Ag NPs 3 against *St. aureus* MDC 5233, *B. subtilis* WT-A17, *E. coli* VKPM-M17, *S. typhimurium* MDC 1754, *E. coli* DH5α-pUC18 and *E. coli* pARG-25, respectively. The experiment was repeated with the same sample (Ag NPs 3) after 1 week, and a significant decrease in the antibacterial activity of Ag NPs 3 was observed whilst the colloidal Ag NPs retained the activity. This decrease in activity could be attributed to the possible aggregation of NPs.

In case of Gram-positive bacterial strains, the activity of colloidal and biosynthesized Ag NPs was almost similar, but in case of Gram-negative strains we had some diversity in the influence—*E. coli* VKPM-M17 was more sensitive to the biosynthesized NPs, compared to the colloidal ones (Fig. 6) (p < 0.01). Ag NPs 3 inhibited the growth of *E. coli* strains even stronger than the standard (Fig. 6) (p < 0.05).

**Fig. 7:**
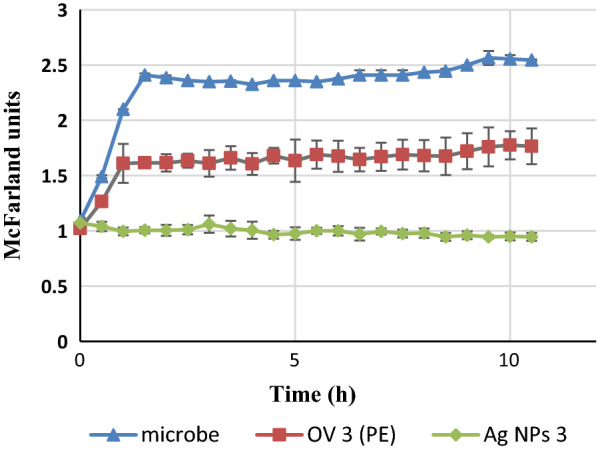
The growth curve of *E. coli* VKPM-M17 compared with the growth curves of bacteria under the influence of OV 3 and Ag NPs 3

The growth kinetics assay was employed to understand the pattern of bacterial growth under the influence of biosynthesized Ag NPs and the related extract. A significant inhibition in growth of *E. coli* VKPM-M17 was observed for both samples *i.e.* extract and the Ag NPs 3 as presented in Fig. 7.

The values of μ_2_ and g_2_ were also calculated for *E. coli* VKPM-M17 treated with OV 3 extract (t_0_ = 0 and t = 1 h). The values of calculated growth rate constants for Ag NPs 3 and OV3 extract were 0.529 h^−1^ and 0.456 h^−1^, respectively. The mean generation time of 1.31 h and 1.52 h was calculated for *E. coli* VKPM-M17 treated with Ag NPs 3 and OV 3 extract, respectively.

## Discussion

Ag NPs synthesized using Oreganum leaves extract have small sizes, they are polidisperse, having different Ag content and tendency to aggregation. Among plants from different regions used to synthesize NPs, Ag NPs 3 (see Materials and methods and Results) were determined with the highest antibacterial activities.

Geographical origin and the climate conditions of plant growth significantly influence the quantitative and qualitative composition of phytochemicals present in plants, as a result, *O. vulgare* leaves extracts exploited in this study exhibited different antioxidant and reducing activities, which led to the synthesis of NPs of varying degrees of stability. Thus, the chemical composition of plant extracts is very important, and by changing it, more stable particles can be synthesized. We suggest further research using separate phyto-molecules instead of plant extracts that contain a mixture of different phyto-molecules. This may help to better understand, which phyto-molecules are more favorable for the green synthesis of Ag NPs.

The antibacterial effect of Ag NPs on Gram-positive and Gram-negative bacterial strains occurs differently since the structure and the charge of the cell wall of these bacteria are varying. Gram-negative bacteria are more susceptible to Ag NPs than Gram-positive bacteria, as described also by different authors (Mnatsakanyan and Trchounian 2018; Aghajanyan et al. 2020).

To explain antibacterial effects, it should be noted that, first of all, Ag NPs damage the cell membrane (Trchounian et al. 2018; Gabrielyan and Trchounian 2019). Moreover, Ag NPs alter the permeability of the cell membrane and disrupt the functioning of the bacterial respiratory chain or the proton F_O_F_1_-ATPase, which are ones of the causes of cell death (Trchounian et al. 2018; Gabrielyan and Trchounian 2019). In addition, Ag NPs induce the formation of reactive oxygen species (ROS) (e.g., superoxide, hydroxyl radicals, singlet oxygen) and inhibit the expression of antioxidant enzymes (i.e., superoxide dismutase and hydroperoxidase), as a result of which the bacterial cell is exposed to oxidative stress and dies (Qing et al. 2018). Ag NPs exhibit a genotoxic effect that causes various damages to DNA sequences. In the presence of Ag NPs, DNA loses its ability to replicate. It can also lead to a malfunction of the repair system (Morones et al. 2005).

The shapes of NPs are also of great importance by the means of influencing on the cell membrane of bacteria. In this regards it would be valuable to mention that so-called green Ag NPs of round-shaped forms are more active, compared to the other forms (Trchounian et al. 2018). Ag NPs obtained by the reduction of plant origin substances extracted from the *O. vulgare* leaves are also possessing round shapes. The similar results have been also described by Aghajanyan et al. (2020), who used as reducing agents the extracts obtained from the *Artemisia annua*. But in contrast to the NPs obtained by using *Artemisia* extracts, the antibacterial activity of NPs obtained by the reduction of AgNO_3_ with the *O. vulgare* leaves extracts possesses 7–9 times higher activity.

In general, the biosynthesis of Ag NPs employing plant extracts offers a simple, inexpensive and safe method, which is widely accepted by several researchers throughout the world (Kumar and Yadav 2009). Some literature data are suggesting the synergistic effect of biologically synthesized Ag NPs and some plant origin substances against different bacterial strains, including multidrug-resistant bacteria, reducing their MIC values and reducing the time of action compared to bio-Ag NPs used alone (Scandorieiro et al. 2016). Biologically synthesized Ag NPs exhibited good antibacterial activity against antibiotic non-resistance as well as antibiotic-resistance bacterial stains, although such particles cannot be utilized for long time due to instability and a tendency to aggregation. Despite this, improving the stability of Ag NPs, these nanoparticles can be used in various biomedical and biotechnological applications (Nakamura et al. 2019).

## Data Availability

All data generated or analyzed duringthis study are included in this published article.
